# Benzo(a)pyrene and Gut Microbiome Crosstalk: Health Risk Implications

**DOI:** 10.3390/toxics12120938

**Published:** 2024-12-23

**Authors:** Intan Rizki Mauliasari, Hee Ju Lee, Song Yi Koo, Emmanuel Hitayezu, Anh Nguyen Thi Kieu, Sang-Min Lee, Kwang Hyun Cha

**Affiliations:** 1 Center for Natural Product Systems Biology, Korea Institute of Science and Technology (KIST), Gangneung 25451, Republic of Korea; intanmauliasari@kist.re.kr (I.R.M.); hjlee81@kist.re.kr (H.J.L.); ninesong2@kist.re.kr (S.Y.K.); 220528@kist.re.kr (E.H.); kieuanh@kist.re.kr (A.N.T.K.); 2 Department of Aquatic Life Medicine, College of Life Sciences, Gangneung-Wonju National University, Gangneung 25457, Republic of Korea; smlee@gwnu.ac.kr; 3 Department of Food Science, College of Life Sciences, Gangneung-Wonju National University, Gangneung 25457, Republic of Korea; 4 Natural Products Applied Science, KIST School, University of Science and Technology, Gangneung 25451, Republic of Korea; 5 Department of Convergence Medicine, Wonju College of Medicine, Yonsei University, 20, Ilsan-ro, Wonju 26493, Republic of Korea

**Keywords:** Benzo(a)pyrene, gut microbiome, dysbiosis, health risk, microplastics

## Abstract

This review delves into the impact of benzo(a)pyrene (B(a)P), which is a toxic and pervasive polycyclic aromatic hydrocarbon (PAH) and known carcinogen, on the human health risk from a gut microbiome perspective. We retrieved the relevant articles on each PAH and summarized the reporting to date, with a particular focus on benzo(a)pyrene, which has been reported to have a high risk of gut microbiome-related harm. B(a)P exposure can compromise the homeostasis of the gut microbiota, leading to dysbiosis, a state of microbial imbalance. The consequences of B(a)P-induced gut dysbiosis can be far-reaching, potentially contributing to inflammation, metabolic disorders, and an increased risk of various diseases. Additionally, due to the strong coupling between B(a)P and microparticles, the toxicity of B(a)P may be further compounded by its reaction with strong gut disruptors such as micro-/nanoplastics, which have recently become a serious environmental concern. This review summarizes current research on the impact of B(a)P on the gut microbiome, highlighting the intricate relationship between environmental exposure, gut health, and human disease. Further research is necessary to elucidate the underlying mechanisms and develop effective strategies to mitigate the adverse health effects of B(a)P exposure.

## 1. Introduction

Research on the health impacts of environmental pollutants has evolved significantly over time. Polycyclic aromatic hydrocarbons (PAHs) are a class of ubiquitous environmental pollutants that pose significant health risks to humans. These persistent organic compounds, formed primarily through incomplete combustion of organic materials, are widespread in both urban and rural environments. PAHs have garnered considerable attention from the scientific community and regulatory bodies due to their potential to cause adverse health effects, even at low exposure levels [1,2]. The ubiquity of PAHs in the environment, coupled with their ability to bioaccumulate in the food chain, makes human exposure nearly unavoidable. Sources of exposure are diverse, ranging from air pollution and occupational settings to dietary intake and tobacco smoke. This widespread presence underscores the critical need for continued research, effective monitoring, and stringent regulatory measures to mitigate the health risks posed by these pervasive contaminants. The health risks associated with PAH exposure are of particular concern. Numerous studies have established strong links between PAH exposure and various forms of cancer, including lung, skin, and bladder cancers [3,4,5]. The International Agency for Research on Cancer (IARC) has classified several PAHs, most notably benzo[a]pyrene (B(a)P), as Group 1 carcinogens, indicating that they are carcinogenic to humans.

The gut microbiome, a complex community of microorganisms in the human gastrointestinal tract, plays a crucial role in various physiological processes, including nutrient absorption, immune system development, and protection against pathogens [6,7]. Disruptions to this complex ecosystem, often referred to as dysbiosis, can have far-reaching implications for human health. Understanding the complex interplay between environmental exposures, the gut microbiome, and human health has become increasingly important in recent years for a variety of toxicants, including heavy metals, persistent organic pollutants, and microplastics (MPs).

**Figure 1:**
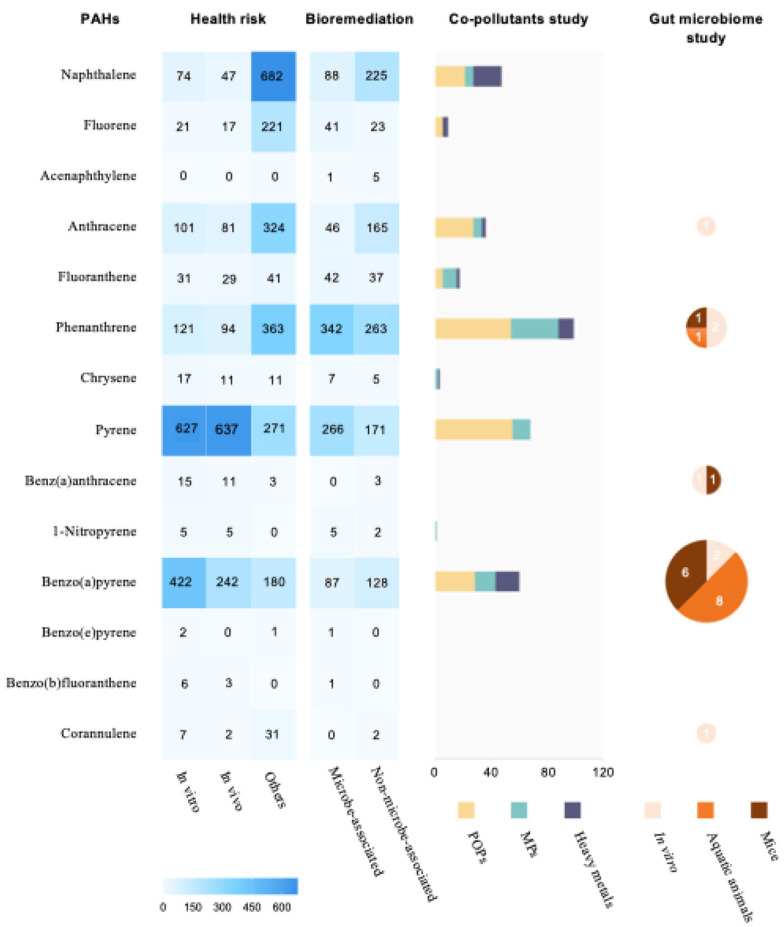
Publication trends in polycyclic aromatic hydrocarbon (PAH) research (to facilitate data interpretation above, the paper numbers were collected from PubMed and Scopus and represented graphically in the heatmap, stack bar, and diagram generated using GraphPad Prism 10.1.2).

Figure 1 illustrates the publication trends in PAH research by 2023, highlighting the research field for various PAH compounds and increasing the focus on specific PAHs such as naphthalene, pyrene, and B(a)P. While other PAHs such as naphthalene, phenanthrene, and pyrene continue to be studied, research on B(a)P has expanded considerably, particularly regarding its impact on the gut microbiome. B(a)P exposure has been shown to induce gut dysbiosis, altering the composition and diversity of the gut microbiota and potentially impairing its functionality [8,9]. While previous B(a)P research characterized the metabolic disturbance, carcinogenic potential, and broader health impacts, recent B(a)P research has shifted its focus from traditional toxicity studies to exploring the gut microbiome’s role in mediating its effects. This emerging research underscores the critical importance of the gut microbiome in shaping our response to environmental pollutants such as B(a)P.

Moreover, oxidative stress, an imbalance between reactive oxygen species production and antioxidant defenses, can significantly impact gut microbiota. Excessive reactive oxygen species (ROS) can damage cellular components, including DNA, proteins, and lipids, potentially disrupting the gut microbiota balance and leading to dysbiosis. The gut microbiota itself plays a role in regulating oxidative stress, producing both ROS and antioxidants. This complex interplay between oxidative stress and gut microbiota is an important area of research, with implications for understanding various health and disease states [10].

Overall, this review delves into the impact of B(a)P exposure on the gut microbiome, exploring the mechanisms by which B(a)P disrupts microbial balance and the potential consequences for human health. We reviewed the effects of B(a)P on gut microbial diversity, composition, and functionality, drawing upon existing research to provide a comprehensive overview of this critical area of study. Understanding the interplay between B(a)P exposure and the gut microbiome is crucial for developing strategies to mitigate the health risks associated with this pervasive environmental pollutant.

## 2. Literature Search and Selection Methods

A thorough literature search was conducted across multiple databases, including PubMed, Scopus, ScienceDirect, Springer Online, and Google Scholar, to identify relevant studies published between 2012 and 2024. The search employed the following keywords: “Benzo(a)pyrene AND health risk AND gut microbiome AND heavy metals AND (microplastics OR nanoplastics)”. We excluded review and research articles that focused on gut microbiome studies but did not specifically address gut microbiome dysbiosis and its potential link to health risks. Over 50 research articles were reviewed to evaluate the effects of B(a)P and co-exposure to environmental pollutants, such as microplastics, on risks through gut microbiome disturbances.

## 3. B(a)P and Its Health Risks

### 3.1. History of B(a)P Health Risk Study

**Figure 2:**
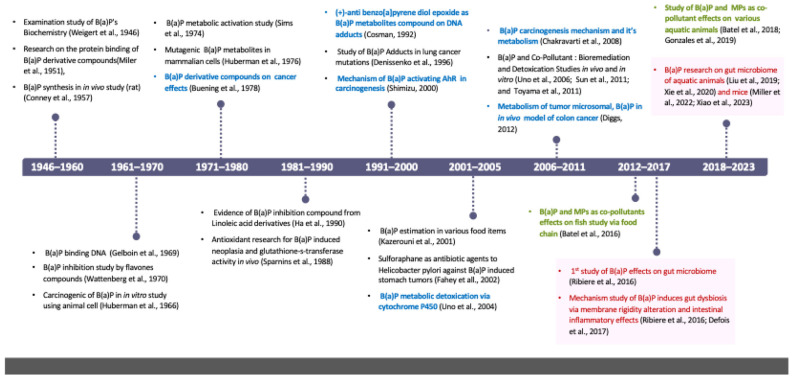
Research history on the health risks of benzo(a)pyrene (B(a)P). Blue text indicates studies on benzo(a)pyrene’s carcinogenic mechanisms, green text represents research on the risks of simultaneous exposure to this substance and microplastics, and red text denotes studies on its effects on the gut microbiome [5,12,15,16,17,18,19,20,21,22,23,24,25,26,27,28,29,30,31,32,33,34,35,36,37,38,39,40,41,42].

Research on B(a)P has a history spanning decades (Figure 2). It was first identified as a component of coal tar as early as 1913 and was scientifically reported in 1945 [11]. As illustrated in Figure 2, from 1946 to the 1980s, scientists established the study of B(a)P biochemistry and metabolic activation, where it becomes highly reactive in the body (in vivo), leading to DNA damage and cancer. In the 1990s, governments and regulatory bodies, such as the U.S. Environmental Protection Agency (EPA), established limits and guidelines for B(a)P exposure in air, water, burnt food, and soil to protect public health and the environment. Ongoing studies have developed biomarkers to measure B(a)P toxicity exposure in individuals, and ongoing research explores establishing a mode of action link between this compound and cancer [5,12]. B(a)P remains a focal point of research in toxicology and environmental science, where scientists are exploring its health hazards, routes of exposure, and strategies to mitigate its effects on human health and aquatic environments. For example, research on combined exposure to MPs and nanoplastics (NPs) in aquatic animals has gained significant attention in recent years due to growing concern about the environmental impact of plastic pollution [13,14,15].

Particularly in recent studies, researchers continue to investigate the extent of contamination, the mechanisms of toxicity, and the long-term ecological implications for the gut microbiome in individual animals, particularly in aquatic animals [43,44] and mice [45]. This area of research is shedding light on the complex interactions between pollutants, microbial communities, and ecological processes, with implications for both environmental health and human well-being.

### 3.2. Toxic Mode of Action of B(a)P

B(a)P undergoes metabolic activation in the human body, which can lead to the formation of highly reactive intermediate metabolites [46]. Briefly, the enzymes in the liver convert B(a)P into reactive intermediates that can covalently bind to DNA, leading to genetic mutations [4]. Once inside the body, B(a)P undergoes metabolic activation, primarily by enzymes such as cytochrome P450 enzymes (e.g., CYP1A1 and CYP1B1), which can convert it into highly carcinogenic reactive intermediates, such as B(a)P-7,8-dihydrodiol-9,10-epoxide (BPDE), in tissues such as the liver and others [47,48]. Reactive intermediates such as BPDE can covalently bind to DNA, forming DNA adducts that distort the standard DNA structure, and these DNA disruptions lead to mutations in critical genes, including tumor suppressor genes and oncogenes [49]. Furthermore, B(a)P exposure can generate reactive oxygen species (ROS) and induce oxidative stress within cells [50,51]. ROS can damage cellular components, including DNA, proteins, and lipids, increasing the risk of oxidative DNA damage and promoting inflammation [52,53,54]. Consequently, this imbalance has been implicated in the pathogenesis of various diseases, such as cancer, cardiovascular diseases, and respiratory disorders.

B(a)P is metabolically activated to a series of reactive intermediates by CYP450 enzymes, particularly CYP1A1 and CYP1B1, under the control of the aryl-hydrocarbon receptor (AhR). There is strong evidence that the benzo(a)pyrene diol epoxide (BPDE) mechanism operates in mouse lung tumorigenesis [55], while there is also strong evidence that both the radical-cation and the diol epoxide mechanisms are involved in mouse skin carcinogenesis [5]. The first step in the action of B(a)P metabolic transformation is the binding of the molecule to the aryl hydrocarbon receptor (AhR), which leads to the activation of CYP1A1 and the formation of reactive intermediates [56,57]. The AhR is a transcription factor that regulates gene expression in xenobiotic metabolisms, such as CYP1A1 and CYP1A2 [58,59]. The oxidation reactions of B(a)P activation are promoted by cytochrome P450s, with CYP1A1 and CYP1B1 exhibiting the highest catalytic specificity towards B(a)P [60,61]. In vitro experiments with recombinant human P450 enzymes from *E. coli* and *Trichoplusia* in cells have shown that CYP1A1 and CYP1B1 exhibit the highest catalytic specificity towards B(a)P [62]. CYP1A1 was considered uniquely responsible for PAH activation until the early 1990s, when CYP1B1 was identified. Thus, factors that influence this balance, including genetic variations in CYP enzymes and the presence of protective antioxidants, can modulate an individual’s susceptibility to B(a)P-induced toxicity [63]. Understanding these metabolic pathways is essential for assessing the health risks associated with B(a)P exposure and for developing strategies to mitigate its toxic effects.

Additionally, mitochondrial unfolded protein response (UPR^mt^) is a critical pathway implicated in various diseases, including metabolic disorders and cancer. Emerging evidence suggests an interesting connection between the UPR^mt^ and the gut microbiota. The gut microbiota plays a vital role in host metabolism and immune modulation, influencing the development of both metabolic disorders and cancer. Certain pathogenic gut bacteria can trigger inflammatory responses and recruit specific immune cells, thereby impacting the progression of these diseases. Some bacterial pathogens release toxins that directly target mitochondria, leading to dysfunction, protein damage, and potential disruption of the mitochondrial membrane. These toxins can induce mtDNA damage and trigger apoptotic pathways [64,65]. Alterations in mitochondrial function, particularly oxidative phosphorylation (OxPhos), are key factors in the development of metabolic diseases such as obesity and insulin resistance. In the context of cancer, the inhibition of key UPR^mt^ regulators, such as ATF5, ATF4, and SIRT, has been linked to cancer growth and progression. ATF5, a retrograde signaling molecule, is overexpressed during UPR^mt^ activation and contributes to cancer cell invasiveness by inducing integrin expression and maintaining OxPhos function. ATF4 enhances the resistance of gastric cancer to chemotherapeutic drugs, such as cisplatin. Additionally, SIRT-mediated UPR^mt^ can promote cancer cell metastasis by preserving mtDNA mutations, thereby maintaining mitochondrial function during metastasis [66]. 

### 3.3. Human Exposure and Epidemiological Research of B(a)P

Some clinical studies have evaluated the actual risk exposure of B(a)P, other PAHs, and other contaminants. The summarized data in Table 1 provide reference concentrations for pollutant compounds, including B(a)P, which refers to the Integrated Risk Information System (IRIS) and the United States EPA. B(a)P is classified as a Group 1 carcinogen by the International Agency for Research on Cancer (IARC) in human adverse-level effect at dose 1 per mg/kg-day. There is substantial evidence supporting its carcinogenicity in humans when compared to other PAHs, such as naphthalene, fluorene, anthracene, fluoranthene, phenanthrene, chrysene, pyrene, benzo(a)anthracene, 1-nitropyrene, and corannulene. Although these other PAHs may still pose health risks, particularly with chronic or high-level exposures, B(a)P remains the most concerning regarding its carcinogenic potential and well-documented health risks. B(a)P’s health risks are primarily associated with inhalation of contaminated air, often found in areas with elevated levels of air pollution, and dietary intake, particularly from grilled or smoked foods and tobacco smoke [63]. When considering their exposure routes closely related to daily exposure for organisms, including humans, B(a)P tends to have higher toxicity effects on health risks compared to other persistent organic pollutants (POPs) and heavy metals such as polychlorinated biphenyls (PCBs) and lead (Pb), which are mainly linked to a lesser extent exposure through inhalation in industrial or specific environmental settings [67].

B(a)P has a substantial impact on epidemiological research, primarily due to its well-established carcinogenic properties and its association with various adverse health outcomes, including cardiovascular diseases, immunotoxicity, reproductive issues, respiratory disorders, gastrointestinal disturbances, and DNA damage, which can lead to the development of cancer [68,69,70]. In the realm of cancer epidemiology, extensive studies have firmly established a strong link between B(a)P exposure and several types of cancer. Interestingly, epidemiological research on B(a)P in the context of the gut microbiome is a relatively emerging area of study, but holds significant potential implications for our understanding of environmental exposures and their effects on human health. Previous studies have revealed significant associations between gut microbiome dysbiosis and the development of cancers like colorectal and lung cancer [71]. Recent research suggests that the gut microbiome has local and systemic effects on cancer disease and that gut dysbiosis is involved in carcinogenesis induced by pollutants and may play a role in the progression of cancer development.

**Table 1:** Exposure levels and legally safe permissible concentrations for PAHs in humans.

Pollutant	Structure	Pollutant Sources	Human Daily Exposure Level	Human Adverse Level Concentration ^1^	
				Carcinogenic	Other Systems
Naphthalene	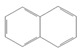	Household and industry	Assumed for 70 kg adult is 1.127 μg/kg per day from air, 0.237 μg/kg per day from food, and 0.235 μg/kg per day from house dust	Probable human carcinogen	Respiratory: 9.3 mg/m^3^ (LOAEL-HEC)
Fluorene	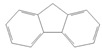	Industry	Not assessed	Not assessed	Not assessed
Anthracene	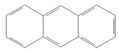	Industry	77.4 ng/m^3^ from the air [72]	Not classifiable as to human carcinogenicity	1.0 × 10^3^ mg/kg-day (NOAEL)
Fluoranthene	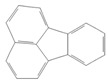	Industry	Not assessed	Not assessed	Hepatic, urinary: 1.25 × 10^2^ mg/kg-day (NOAEL)
Phenanthrene	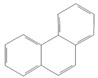	Industry	Not assessed	Not classifiable as to human carcinogenicity	0.1–0.2 mg/m^3^ for airborne exposure limit
Chrysene	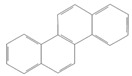	Industry	Not assessed	Probable human carcinogen	Not assessed
Pyrene	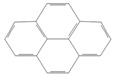	Industry	Not assessed	Not classifiable as to human carcinogenicity	Urinary: 7.5 × 10 mg/kg-day (NOAEL)
Benz(a)anthracene	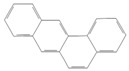	Industry	Not assessed	Probable human carcinogen	Not assessed
1-Nitropyrene	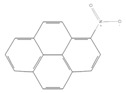	Industry	Not assessed	Not assessed	Not assessed
Benzo(a)pyrene	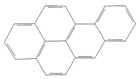	Household and industry	52 to 95 ng/cigarette, 7.20 ± 1.11 μg/m^3^ in the air, 4.15 μg/kg in well-done steaks, and 4.00 to 8.33 ng/L in drinking water [63]	1 per mg/kg-day	Embryo: 4.6 × 10^3^ mg/m^3^ (LOAEL)
Corannulene	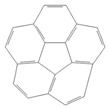	Lab- synthesized chemical	Not assessed	Not assessed	Not assessed
Polychlorinated biphenyl (PCBs)	-	Industry	For adult exposure: 3.04 ng/kg-day indoor inhalation, 3.0 ± 2.2 ng/kg-day for dietary intake [73]	1 µg/m^3^ (10-h time-weighted average)	Not assessed
Lead (Pb)	-	Household and industry	Human daily intake from food: 0.1 to 0.3 µg/kg body weight/day [74]	Not assessed	Brain–intelligence: Blood lead level of <10 μg/dL in child study [75]

^1^ All human adverse-level concentration references were retrieved from the Integrated Risk Information System (IRIS) or Environmental Protection Agency (EPA) database; not assessed, and no references were found.

## 4. B(a)P and Gut Microbiome Interaction

The gut microbiome, a complex community of microorganisms in the gastrointestinal tract, plays a vital role in human health, including digestion, immune system regulation, and metabolic balance. Recent studies have explored how B(a)P exposure impacts the composition and diversity of this microbiome, potentially affecting its functionality and human health outcomes. The effects of B(a)P exposure on gut microbiome are summarized in Table 2.

### 4.1. B(a)P Exposure and Gut Dysbiosis

According to Table 2, although recent in vitro studies directly examining the relationship between B(a)P-induced gut microbiome dysbiosis and human health risks remain limited, existing research indicates that B(a)P exposure can alter the composition of the gut microbiome. These changes affect the volatile organic compounds or known as volatolome, which is linked to metabolic diseases. This suggests a potential pathway through which B(a)P exposure may contribute to metabolic disorders [18] and causing inflammatory bowel disease (IBD) through its impact on gut microbiome (discussed further in Section 4.2). Rodent studies have shown that B(a)P exposure leads to significant alterations in gut microbial composition, resulting in reduced alpha diversity. Ribiere et al. demonstrated that 28-day exposure to B(a)P (10 mL/kg BW) in murine models induced gut microbial shifts, decreasing *Bacteroides*, *Parabacteroides*, and *Paraprevotella*. Furthermore, He reported that long-term oral exposure of B(a)P at 50 mg/kg mice BW for 42 days induced gut microbiome alteration, especially the genera *Faecalibaculum*, *Lactobacillus*, *Acinetobacter*, *Desulfovibrio,* and *Alistipes,* which could induce colon inflammation. Similar to Du et al., the study evaluated long-term exposure in mice via oral gavage at low-dose B(a)P (50 u µg/mouse/day) for 23 days, resulting in gut dysbiosis, characterized by an increased abundance *of Lachnospiraceae bacterium 3-2*, *Lachnospiraceae bacterium COE1*, and *Prevotella* sp. *MGM1*, which is linked to allergic responses [45].

Studies on aquatic animals, including fish, scallops, and sea cucumbers, have shown that B(a)P exposure decreases alpha diversity while increasing the abundance of Fusobacteria, Proteobacteria, and Bacteroidetes [80,81,83]. These effects were observed through various exposure routes, including water exposure, feeding manipulation, and intraperitoneal injection. According to those studies, B(a)P and other environmental contaminants can exert a substantial influence on the gut microbiota, with their impact primarily contingent on the long duration and low concentration intensity of the host’s exposure to pollutants. These findings indicate a potential connection between B(a)P-induced gut dysbiosis and health risks. In particular, translational studies that bridge the gap between in vitro and in vivo research on human studies are needed to fully understand the health implications of B(a)P exposure. The current in vitro evidence provides a strong basis for exploring the effects of B(a)P on the gut microbiome in more advanced in vivo models and eventually human populations. This will require carefully designed human studies that functionally evaluate both B(a)P exposure and gut microbiome composition alongside key metabolic health markers. Such research is crucial to determine the true impact of B(a)P on human health and to inform strategies for minimizing potential risks.

### 4.2. Key Influential Factors for B(a)P-Induced Gut Microbiome Change

The B(a)P-associated gut microbiome changes may be linked to the lipophilic characteristics of B(a)P. As lipophilic substances, PAHs such as B(a)P can permeate the cytoplasmic membrane, increasing membrane fluidity and damaging bacterial cells, leading to bacterial death and an unbalanced gut composition [18,84]. The lipophilic nature of B(a)P influences its interaction with both Gram-positive and Gram-negative bacteria, although the structural differences between these bacteria types lead to some variations in their effects. In Gram-negative bacteria, B(a)P lipophilicity allows it to readily penetrate the outer membrane, which is rich in lipopolysaccharide. This can disrupt the outer membrane structure, potentially leading to the release of LPS, a potent endotoxin that triggers inflammation [85]. Furthermore, B(a)P can cross the inner membrane, affecting its fluidity and potentially damaging essential membrane proteins [86]. The periplasmic space, located between the outer and inner membranes, contains peptidoglycan, which can also be affected by B(a)P exposure, potentially triggering signaling through NOD receptors [87]. Despite lacking an outer membrane, Gram-positive bacteria possess a thick peptidoglycan layer external to their single-cell membrane. B(a)P’s lipophilic nature still allows it to penetrate this peptidoglycan layer and reach the cell membrane, where it can disrupt membrane fluidity and function, similar to its effects on Gram-negative bacteria. The absence of an outer membrane and LPS in Gram-positive bacteria means that B(a)P exposure will not lead to LPS release and the associated inflammatory effects seen in Gram-negative bacteria [88]. In both cases, B(a)P’s lipophilicity enables it to interact with and disrupt bacterial membranes, ultimately affecting cell viability and potentially contributing to gut dysbiosis.

Moreover, volatile organic compounds (VOCs) generated by the gut microbiota have been examined as potential contributors to pollutant-induced gut dysbiosis [18,89]. Six distinct VOCs, including benzaldehyde, 3-octane, 2-pentyl furan, butyl butanoate, 2-methyl-phenol, and 2-hexyl furan, were significantly detected in human fecal bacteria after a 24 h in vitro exposure [18]. De et al. observed shifts in metabolites within the benzenoid, ketone, and furan derivative categories, likely attributable to disruptions in typical bacterial ecology, particularly in pathologies such as inflammatory bowel disease (IBD) [90].

Sex differences in gut microbiota have been reported in B(a)P exposure studies using fish and mice as in vivo models. In juvenile fathead minnows, the gut microbiota community structure was notably changed in female fish but not in male fish after exposure to a low B(a)P dose [91]. These distinctions are ascribed to hormonal regulation, particularly the influence of androgen receptors. The gut microbiota controls estrogen levels by producing β-glucuronidase, which converts estrogens into active forms. Microbiota imbalance can disrupt this process, ultimately reducing circulating estrogen levels. Alterations in circulating estrogen levels can contribute to metabolic inflammation, leading to various diseases [92,93,94]. Beibei et al. demonstrated that oral administration of B(a)P in female mice led to increased susceptibility to allergic symptoms compared to male mice, associated with increases in specific bacterial species [45]. Although clinical studies comparing B(a)P exposure effects between males and females are limited, it is evident that pollutant exposure can disrupt the gut microbiome in a sex-specific manner, possibly influenced by hormonal regulation.

### 4.3. Microbiome-Related Health Risks of B(a)P

B(a)P has been shown to accumulate within the intestinal tract, resulting in significant alterations in the intricate balance of the gut microbiome. This intricate microbial ecosystem plays a pivotal role in maintaining human health, significantly influencing the development of various chronic diseases. These health conditions encompass a spectrum of disorders, ranging from localized intestinal issues such as colonic injury [33,77,95] to systemic diseases, including carcinogenic effects like lung cancer and colorectal cancer, cytotoxic responses linked to cancer, xenobiotic metabolism, and modulation of allergic responses [6,96].

#### 4.3.1. Colonic Injury and Immunity Impairment

Sub-chronic oral exposure to B(a)P resulted in moderate inflammation, primarily affecting the ileal mucosa. In a murine study, this pollutant exposure led to changes in fecal and mucosa-associated microbiota composition [33]. Specifically, it reduced beneficial bacteria associated with short-chain fatty acids (SCFA), such as *Clostridium* and *Coprococcus*, from the *Lachnospiraceae* family. Conversely, there was an increase in the abundance of pro-inflammatory bacteria, such as *Paraprevotellaceae*, *Turicibacter*, and *Desulfovibrionaceae*, which are associated with colitis. Notably, *Desulfovibrionaceae* produces endotoxins and toxic sulfur, which can contribute to immune disorders and colon damage. A supporting study reported that B(a)P exposure in mice elevated metabolic pathways related to lipopolysaccharides (LPS) and sulfur compounds, along with increasing the presence of *Desulfovibrionaceae* [77]. Specific metabolites or elements of intestinal flora can cause immune cells to produce cytokines, which can modify the immunological response [97,98]. B(a)P treatment increased the prevalence of the pathogenic genus *Staphylococcus*, which is linked to increased inflammation and decreased immunomodulatory function of the intestines at the genus level [99]. Du et al. found that in mice exposed to intestinal allergic inflammation, the levels of inflammatory cytokines were linked to the presence of specific bacterial taxa, particularly *Lachnospiraceae bacterium* 28-4 and *Alistipes inops* [45]. This was paralleled by elevated allergic cytokines and intestinal permeability. The abundance of *L. bacterium* 3-2, *L. bacterium* COE1, and *Prevotella* sp. MGM1 positively correlated with the exacerbation of intestinal inflammation. These results suggest a correlation between inflammatory responses and specific bacterial taxa, indicating their potential role in gut health and immune response. A key area of concern is the link between environmental pollutants, chronic inflammation, and the loss of self-tolerance in autoimmune diseases. Pollutants can disrupt the gut microbiome, leading to dysbiosis and increased intestinal permeability. This can allow harmful substances, including bacterial components such as lipopolysaccharides, to enter the bloodstream, triggering systemic inflammation [100]. Chronic inflammation, in turn, can contribute to the breakdown of self-tolerance, where the immune system mistakenly attacks the body’s own tissues, characteristic of autoimmune diseases. Environmental factors, including xenobiotics, play a significant role in the development of autoimmunity, often acting as triggers in genetically susceptible individuals [101]. Further research is needed to elucidate the precise mechanisms by which pollutants contribute to autoimmunity in humans. Longitudinal studies assessing pollutant exposure, gut microbiome composition, inflammatory markers, and autoimmune disease development are crucial. This research will inform targeted interventions to mitigate the impact of environmental pollutants on human health, particularly in preventing or managing autoimmune diseases.

#### 4.3.2. Carcinogenic and Xenobiotic Implications

In cancer research, the occurrence of particular bacterial strains (including *Bifidobacterium*, *Intestinimonas*, *Alistipes*, *Odoribacter*, and *Acetatifactor*) and the activation of specific metabolic pathways (including linoleic acid metabolism, unsaturated fatty acid production, and steroid hormone biosynthesis) were linked with the gut–lung axis correlation in a mouse model of lung cancer [71]. The gut microbiota can modulate the immune response in the lungs through various mechanisms, such as the activation of immune cells by T cells producing IL-17 and the production of cytokines and chemokines essential for regulating immune responses in the respiratory system [102,103,104]. Wu reported that B(a)P-induced colon cancer in mice showed significantly increased levels of *Sphingobacteria*, *Gamma-proteobacteria*, and *Lactobacillales*. These bacteria might help transform B(a)P into reactive metabolites that undergo enterohepatic circulation as they interact with gut microbiota, contributing to the reintroduction of reactive xenobiotic metabolites in the intestine [96]. Along with xenobiotic studies, Gentao et al. conducted the intraperitoneal exposure route B(a)P to rats at 100 mg/kg BW and showed there was a notable rise in the hepatic transcriptomic levels of carcinogenic biomarkers associated with xenobiotic metabolism influenced by the gut microbiome [78].

#### 4.3.3. Metabolic Disturbance

Metabolic syndrome (MetS) is a risk factor for developing several complex human diseases, including type 2 diabetes, obesity, cardiovascular disorders, and cancer [7]. While specific studies on B(a)P’s effects on metabolic syndrome related to the gut microbiota are preliminary, previous research has shown that gut microbiota dysbiosis can lead to metabolic syndrome and is involved in bile acid metabolism, glucose, and energy homeostasis [105]. B(a)P exposure altered gut microbiome composition by decreasing *Akkermansia muciniphila* in murine models [33] and elevating the *Firmicutes*/*Bacteroidetes* ratio in various aquatic species. In mice treated with B(a)P, the abundance of *Prevotella* spp. was found to be significantly increased [45]. Dysbiosis of some colonizing bacteria, including *Prevotella* spp., has been associated with type 1 diabetes (T1D) [106]. Additionally, a higher *Firmicutes*/*Bacteroidetes* ratio, similar to that observed in obese animals or those fed a high-fat diet, is linked to metabolic disorders and weight gain [107,108]. This observation might be explained by the fact that *Firmicutes* are more efficient as an energy source than *Bacteroidetes*, resulting in more effective calorie absorption and subsequent weight gain.

## 5. Synergistic Toxic Effects of B(a)P and MPs

### 5.1. MPs as Vectors for B(a)P: Mechanisms, Environmental Fate, and Biological Impacts

The interactions between B(a)P and microplastics (MPs) are of growing concern in environmental science due to their potentially synergistic effects on ecosystems and human health [109]. MPs can act as vectors for contaminants such as B(a)P, disrupting gut microbiomes and causing various indirect hazards to organisms [110,111,112,113,114]. They have considerable adsorption capacity and can transport pollutants over long distances [115,116]. Shaoyong et al. found that B(a)P-loaded aged polystyrene MPs (PS-MPs) promote colonic barrier injury in mice, leading to increased permeability and inflammation mediated by oxidative stress-induced activation of the Notch signaling pathway [117]. The interaction between PS-MPs and B(a)P in acidic environments leads to excessive reactive oxygen species (ROS) production, causing synergistic toxic effects [118,119].

MPs can be ingested by aquatic animals, particularly fish, making them as suitable model organisms for studying the combined effects of MPs and B(a)P on health risks [120]. As MPs can adsorb and carry chemicals, including B(a)P, and deliver them to the host, simultaneous exposure to MPs and B(a)P may adversely affect organisms, particularly aquatic animals, as summarized in Table 3. The interaction between MPs and B(a)P is related to the binding affinity between these two substances. MPs can have varying binding affinity to B(a)P depending on the MP types, such as polystyrene, polyethylene, and polypropylene, and their sizes [121]. MPs/NPs have a strong binding affinity, which enables extensive interactions between them and the surrounding substances [122]. A study on mussels found that B(a)P concentrations increased over time, especially when sorbed to smaller MPs, and that MPs with sorbed B(a)P were more toxic than MPs alone [15]. The mobility of MPs depends on their size, with smaller MPs (<1 mm) showing high mobility in horizontal and vertical directions [123]. Smaller MPs are more mobile than larger MPs, which means that they can more easily penetrate biological barriers and accumulate in tissue, increasing their potential for toxicity [101,107,108].

PS-MPs have a higher binding affinity to B(a)P than other MPs, which might be attributed to their chemical properties. The PS-MP characteristics are also strongly influenced by the presence of the pendant phenyl (C6H5) groups, as the phenyl rings prevent the chains from rotating around the carbon–carbon bonds and give the polymer its well-known stiffness [108]. Another study also stated that the hydrophobicity of PS-MPs is due to its aromatic ring structure, which allows it to interact with other aromatic compounds, such as B(a)P [109,110,111]. Thus, the accumulation of MPs within cells can disrupt cellular functions and potentially lead to cytotoxicity. These findings emphasize the potential for the combined pollutants to exert substantial toxic effects on biological ecosystems.

### 5.2. Combined Effects of B(a)P and MPs on Gut Microbiome

The co-exposure of B(a)P and MPs potentially has more adverse effects on the gut microbiota compared to single exposure to B(a)P because the gut dysbiosis caused by MP exposure has been consistently reported in recent years. Exposure to MPs can cause changes in bacterial species, richness, beta diversity, and gut microbiota composition in various aquatic species, often reducing beneficial bacteria and increasing potential pathogens [133]. Usman et al. found that PS-MP exposure in Javanese medaka fish altered gut flora and caused metabolic abnormalities related to energy metabolism [134]. Also, recent studies have shown that MPs, particularly NPs, can affect the gut–brain axis by altering gut microbiota, intestinal barrier permeability, oxidative stress, inflammation, neurotoxicity, and behavioral disturbances in mice [135,136]. Yang et al. revealed that oral administration of NPs to mice influenced brain function by stimulating macrophage IL-1 signaling in the intestine [137]. Besides, MP ingestion in rats resulted in gut microbiome alterations at the family level (*Muribaculaceae*, *Oscillospiraceae*, *Bacteroidaceae*, *Neisseriaceae*, *Prevotellaceae*, and *Veillonellaceae*), affecting lipid metabolism and potentially impacting brain function and anxiety-related behaviors [138].

Although there are no direct studies of the combined effects of B(a)P and MPs on the gut microbiome, co-exposure to MPs and B(a)P may potentially induce gut microbiota dysbiosis by damaging the intestinal epithelium’s mucus layer, increasing intestinal permeability, and disrupting the gut microbiota barrier. This disruption can lead to changes in microbial diversity, composition, and metabolite profiles, as well as intestinal inflammation. While research in this field is still ongoing, studies examining the impact of MPs on gut microbiota have revealed an emerging and increasingly important area of research [139,140]. These findings highlight the potential health risks associated with exposure to B(a)P and MPs and the need for further research to fully understand the mechanisms underlying their toxic effects on the onset of various diseases linked with the gut microbiome in aquatic animals, humans, and other animals.

**Figure 3:**
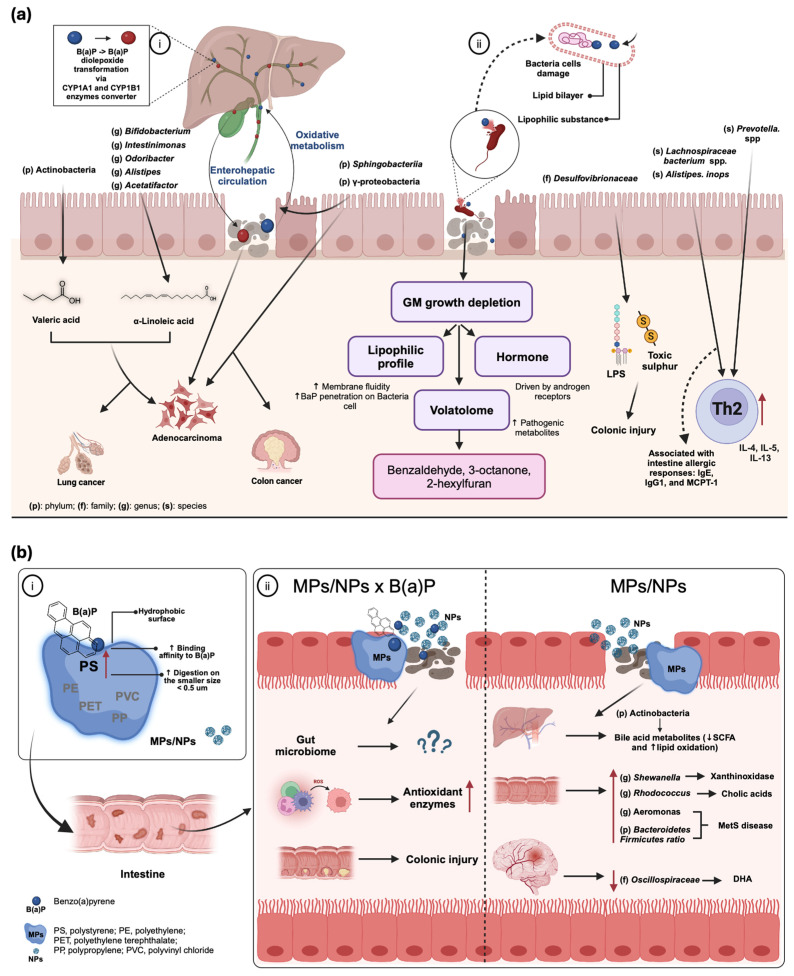
(**a**) A schematic illustration depicting the metabolic pathway of B(a)P (**i**) elucidating its enterohepatic circulation and association with cancer development involving diverse bacterial interactions and (**ii**) delineating its toxicity, which induces damage to bacterial cells, subsequently contributing to immune disorders and resultant diseases. (**b**) Illustration of (**i**) several types of MP/NP characteristics on combined adsorption with B(a)P and (**ii**) mechanism of micro-/nanoplastic (MP/NP) co-exposure with B(a)P and single exposure of MPs/NPs on gut microbiome disturbances which induces reactive oxygen species (ROS) production, increasing antioxidant activity and causing several chronic diseases in different organs. Yet the co-exposure MPs and B(a)P study effects on gut microbiota require further research, this co-exposure may also damage gut microbiome cells potentially leading to dysbiosis.

Research on B(a)P’s impact on the gut microbiome reveals both direct and indirect health effects, presenting a multifaceted risk profile (Figure 3).

## 6. Conclusions

In conclusion, B(a)P exposure can disrupt gut microbiota balance, leading to dysbiosis that can trigger gut inflammation and influence the host’s immune response, potentially promoting cancer development. Also, gut microbial dysbiosis due to B(a)P exposure may also aggravate metabolic processes, contributing to disorders such as obesity and insulin resistance. However, further in-depth analysis is needed to fully elucidate these complex relationships. Certain gut bacteria can metabolize B(a)P into toxic compounds, and shifts in microbiome composition may alter the balance of these metabolites, impacting cancer risk. Also, due to the strong reactivity of B(a)P with microparticles, B(a)P can act as a carrier of microplastics by binding to them, which have recently been demonstrated to be hazardous to the gut microbiome. As microbiome-related B(a)P research is still limited to several in vitro and in vivo studies, more approaches at different concentrations and treatment periods are needed, as well as combination studies with various microplastic materials and sizes. Most importantly, human studies will be required to determine whether the various B(a)P exposure levels result in differences in human gut microbiome.

## Figures and Tables

**Table 2:** B(a)P exposure effects on alterations in the gut microbial community.

Species Target	Life Stage	Co-Exposure Pollutant	Pollutants Concentration	Exposure Type	Exposure Period	Intestinal Impacts	Ref.
In vitro							
Human microbiome	-	-	0.005, 0.05, and 0.5 mg/mL	Human fecal culture in bio-fermenter	24 h	↓ Microbial volatile in a dose-dependent manner ↓ Transcript level of bacterial chemotaxis toward simple carbohydrate pathways	[18]
Camel rumen and rectum	-	-	50 mg/L	Rumen and gut microbes culture	20 days	*Klebsiella* sp., *Ochrobactrum* sp., and *Bacillus* sp. showed particular function as B(a)P degradation	[76]
In vivo (mice)							
C57BL/6 mice	5-week-old (20–25 g)	-	10 mL/kg B.W.	Oral gavage	28 days	↑ *Bacteroides*, *Parabacteroides*, and *Paraprevotella* >27 days *↓ Lactobacillus, A. muciniphila,* and *Verrumicrobiaceae*	[33]
BALB/c mice treated with Isoorientin	5-week-old	-	50 mg/kg BW	Oral gavage	42 days	*↑ Desulfovibrio*, *Acinetobacter*, *Odoribacter*, and *Veillonella* in B(a)P group ↑ *Faecalibaculum* and *Lactobacillus* in B(a)P + ISO group	[77]
C57BL/6 mice and male Sprague Dawley (S.D.) rats	8-week-old mice (22 ± 2 g) and (220–250 g) rats	Corannulene	100 mg/kg BW COR or B(a)P	Oral gavage and intraperitoneally injection	3 days	↑ *Bacteroidetes* after I.P. injection of B(a)P and COR ↓ *Bacteroidetes* after oral gavage B(a)P and COR *Actinobacteria* ↓ by oral COR but ↑ by oral B(a)P	[78]
Conventional C57BL/6NTac and germ-free C57Bl/6GFTac mice	7-week-old	1-Nitropyrene	180 mg/kg BW./day B(a)P	Oral gavage	72 h	Active P450s enzyme in the liver is impacted by the presence of the gut microbiome, which is modified by PAH metabolism	[79]
C57BL/6 mice (SPF) treated with Ovalbumin	5–6 weeks old	-	50 ug/ mouse/ day	Oral gavage	23 days	↑ *B. virosa* and *N. subflava*, and ↓ *B. uniformis* and *L. bacterium COE1* in B(a)P group ↑ *L. bacterium 3-2*, *L. bacterium COE1*, and *Prevotella* sp. *MGM1* in OVA group	[45]
In vivo *(Aquatic animals)*							
Female and male Fathead minnow *(Pimephales promelas)*	Adult (two years)	-	1.3, 4.0, and 12.0 mg/L	Water immersion	4 days	↑ Alpha diversity in the female group compared to the male group ↓ *Vibrionaceae*, the only abundant family in the male group	[44]
Fathead minnow *(Pimephales promelas)*	Juvenile (2.5-month-old)	-	1, 10, 100, or 1000 u µg/g in food (DM)	Feeding	2 weeks	↓ Alpha diversity, ↑ pathogenic taxa (*Erysipelotrichaceae*, *Moraxellaceae*, and *Caulobacteraceae)*	[80]
Nile tilapia *(Oreochromis niloticus)*	Juvenile (125.6 ± 41.4 g)	-	20 mg/kg B.W.	Intraperitoneal injection	24, 72, and 120 h post-injection	*↑ Fusobacteria* and *Bacteroidetes* in <24 h *↓ Proteobacteria* and *Spirochaetae* in >24 h GM recovered after 72 h and was stable at 120 h post-injection	[81]
Zebrafish* (Danio rerio)*	Embryos (9 days post fertilization)	-	1, 5, and 10 μM	Embryos Incubation test—with different dissection methods	9 days	Gut microbiota significantly altered based on dose-dependent	[82]
Scallop *(Chlamys farreri)*	(5.7 ± 0.3 cm in length)	-	0, 0.4, 2 and 10 μg/L	Water immersion	7, 14 and 21 days	↓ Alpha diversity, ↑ pathogenic bacteria *Mycoplasma* and *Tenacibaculum.* Hydrocarbon-degrading bacteria were found: *Pseudomonas*, *Polaribacter*, *Amphritea*, and *Kordiimonas*	[83]
Sea cucumbers *(Apostichopus japonicus)*	Juvenile (5.36 ± 0.14 g)	-	0, 0.5, 5, and 25 μg/L	Water immersion	14 days	↑ Ratio *of Bacteroidetes* to *Firmicutes*	[43]

**Table 3:** B(a)P and MP co-exposure effects on aquatic animals.

Species Target	MPs Type	MPs Size	MPs Binding Affinity ^1^	Pollutant Concentrations and Exposure Method	Exposure Period	Toxicity Effects	Ref.
Marine mussels (*M. galloprovincialis*)	Green fluorescent polystyrene	10 µm	***	5.5 µg/L MPs + 0.1 µg/L B(a)P in water immersion	5 days	↓ mRNA expression of NF- κB in gills ↑ strong affinity adsorption of B(a)P to PS-MPs ↓ The uptake and toxicity of B(a)P	[124]
Clam (*Scrobicularia plana*)	Low-density polyethylene	11–13 µm	-	MPs with B(a)P adsorbed at one mg/L in water immersion	14 days	Changes in protein expression of the cytoskeleton, cell structure, oxidative stress, energy metabolism, and DNA binding also induce changes in glucose metabolism, RNA binding, and apoptosis	[125]
Marine mussels (*M. galloprovincialis*)	Environmental mixture MPs: polyethylene, polyethylene terephthalate, polypropylene, polyethylene vinyl acetate, and high-density polyethylene	<100 µm	-	50 µg/L MP + 1 µg/L B(a)P in water immersion	1 and 3 days	Induced the apoptosis process: ↑ DNA ligase on day 1 ↑ Bax, Cas-3, and P53 and on day 3 ↓ Bcl-2 and DNA ligase on day 3	[126]
Clam (*Scrobicularia plana*)	Low-density polyethylene	4–6 μm and 20–25 μm	-	1 mg/L MP + 16.64 ± 87 µg/g B(a)P in water immersion	7 and 14 days	4–6 μm-sized MPs resulted in more significant alterations in oxidative stress biomarkers	[127]
Marine mussels (*M. galloprovincialis*)	Polystyrene pristine	4.5 and 45 µm	***	(0.05, 5, 50 µg/L MPs) + 252.3 µg/L B(a)P and Cd	3 days	↑ PS shows a higher affinity to B(a)P than Cd MPs and B(a)P group induced histological alteration in digestive glands	[128]
Brine shrimp larvae and zebrafish embryos	Polystyrene	50 and 500 nm	***	0.069–6.87 mg/L PS + 0.1–10 mg/L B(a)P	24–48 h	↑ malformation prevalence in the highest concentration of MPs and B(a)P groups in zebrafish; meanwhile, NPs were successful vectors to B(a)P in brine shrimp	[129]
White seabass	Polystyrene	2.00–2.83 mm	***	100 g/2 L PS + 1µg/L B(a)P and single dose of 1 µg/L B(a)P and 252 µg/ B(a)P in water immersion	5 days	Single dose of 252 µg/ B(a)P group; fish exposed to polystyrene B(a)P-absorbed polystyrene show significant variations in the observed cellular or behavioral parameters compared to the control group	[130]
Seaworm (*Hediste diversicolor*)	Environmental mixture MPs: polyethylene, polyethylene terephthalate, polypropylene, polyethylene vinyl acetate, and high-density polyethylene	>3, 3.0–1.22, 1.22–0.45 µm	-	1 mg/kg (sediment) of environmental MPs + 1 μg/kg (sediment) B[a]P	3 and 7 days	↑ cytotoxic and genotoxic damage in the co-exposure and single groups after 7 days.	[131]
Marine diatom (*Chaetoceros muelleri*)	Polyethylene terephthalate	-	-	200 mg/L PET + (10, 150 µg/L B(a)P) in medium culture	1, 5, 15 days	↑ SOD and MDA on day 1 and 5 ↓ SOD and MDA on day 15 MPs and 10 µg/L B(a)P group showed higher antagonistic effects to the marine diatom.	[132]

^1^ Microplastics’ binding strength to pollutants, measured by sorption tests, is indicated (-, no sorption test was conducted; ***, the strong binding affinity when tested).

## Data Availability

Data for the results presented in this article will be available upon request.
